# Kinin B_2 _receptor regulates chemokines CCL2 and CCL5 expression and modulates leukocyte recruitment and pathology in experimental autoimmune encephalomyelitis (EAE) in mice

**DOI:** 10.1186/1742-2094-5-49

**Published:** 2008-11-05

**Authors:** Adriana C Dos Santos, Ester Roffê, Rosa ME Arantes, Luiz Juliano, Jorge L Pesquero, João B Pesquero, Michael Bader, Mauro M Teixeira, Juliana Carvalho-Tavares

**Affiliations:** 1 Department of Physiology and Biophysics, Federal University of Minas Gerais, Belo Horizonte, MG, Brazil; 2 Department of Biochemistry and Immunology, Federal University of Minas Gerais, Belo Horizonte, MG, Brazil; 3 Department of Pathology, Federal University of Minas Gerais, Belo Horizonte, MG, Brazil; 4 Escola Paulista de Medicina, São Paulo, SP, Brazil; 5 Max Delbruck Center for Molecular Medicine, Berlin, Germany

## Abstract

**Background:**

Kinins are important mediators of inflammation and act through stimulation of two receptor subtypes, B_1 _and B_2_. Leukocyte infiltration contributes to the pathogenesis of autoimmune inflammation in the central nervous system (CNS), occurring not only in multiple sclerosis (MS) but also in experimental autoimmune encephalomyelitis (EAE). We have previously shown that the chemokines CCL2 and CCL5 play an important role in the adhesion of leukocytes to the brain microcirculation in EAE. The aim of the present study was to evaluate the relevance of B_2 _receptors to leukocyte-endothelium interactions in the cerebral microcirculation, and its participation in CNS inflammation in the experimental model of myelin-oligodendrocyte-glycoprotein (MOG)_35–55_-induced EAE in mice.

**Methods:**

In order to evaluate the role of B2 receptor in the cerebral microvasculature we used wild-type (WT) and kinin B2 receptor knockout (B_2_^-/-^) mice subjected to MOG_35–55_-induced EAE. Intravital microscopy was used to investigate leukocyte recruitment on pial matter vessels in B_2_^-/- ^and WT EAE mice. Histological documentation of inflammatory infiltrates in brain and spinal cords was correlated with intravital findings. The expression of CCL5 and CCL2 in cerebral tissue was assessed by ELISA.

**Results:**

Clinical parameters of disease were reduced in B_2_^-/- ^mice in comparison to wild type EAE mice. At day 14 after EAE induction, there was a significant decrease in the number of adherent leukocytes, a reduction of cerebral CCL5 and CCL2 expressions, and smaller inflammatory and degenerative changes in B_2_^-/- ^mice when compared to WT.

**Conclusion:**

Our results suggest that B_2 _receptors have two major effects in the control of EAE severity: (i) B_2 _regulates the expression of chemokines, including CCL2 and CCL5, and (ii) B_2 _modulates leukocyte recruitment and inflammatory lesions in the CNS.

## Background

Bradykinin (BK) and its biologically active metabolites are the functional components of the kallikrein-kinin system. Kinins elicit a wide range of physiological effects including relaxation of vascular smooth muscle in arteries and arterioles, expression of adhesion molecules, leukocyte infiltration, formation of interendothelial gaps and protein extravasation from post-capillary venules, and pain transmission mechanisms [1,2]. The actions of kinins are mediated through stimulation of two subtypes of seven-transmembrane-domain G-protein-coupled receptors, namely B_1 _and B_2_. The B_2 _receptor is constitutively expressed in various cell types, including endothelial cells, nerve fibers, leukocytes and mast cells [3,4]. The B_1 _receptor is generally expressed at low levels under normal conditions but is up-regulated by cytokines in stressful situations, such as shock and inflammation [5-7]. Most of the physiological actions of kinins are believed to be mediated by stimulation of B2 receptors [8].

Experimental autoimmune encephalomyelitis (EAE) is an inflammatory disease of the CNS mediated by CD4+ Th1 cells that serves as experimental model of human multiple sclerosis (MS). A pathological hallmark of MS is infiltration of immune cells across the blood-brain barrier into the CNS causing myelin destruction and axonal injury [9]. It is thought that inappropriate leukocyte recruitment and activation in the brain results in disease symptoms and progression [10]. Thus, reduction of the migration of immune cells into the CNS is a relevant novel therapeutic strategy for the treatment of MS.

Although the potential role of the kinin system on leukocyte entry into the CNS in MS remains unclear, it has been shown that BK can interfere with the mechanism of leukocyte recruitment in various tissues [11]. BK may enhance the expression of adhesion molecules on endothelium cells [12]. BK antagonists reduce leukocyte-endothelium interactions after diverse inflammatory conditions, including global cerebral ischemia [4], leukocyte infiltration in murine mesenteric post-capillary venules [13] and lung inflammation in guinea pigs [14]. BK could potentially modify leukocyte recruitment by production of chemoattractant molecules, such as chemokines. For example, treatment with bradykinin receptor antagonists has been shown to reduce production of chemokines, including KC and MCP-1, after intestinal ischemia and reperfusion [15]. Several studies, have clearly demonstrated the relevance of chemokines for the recruitment of leukocytes into the brain of EAE mice [16-18].

Cross talk between cytokines and kinin receptors has been extensively investigated over the last several years [19-21]. Studies have demonstrated that pro-inflammatory cytokines regulate B_1 _and B_2 _receptor expression [11,22,23] and, conversely, blockade of kinins receptors modifies expression of cytokines and chemokines [24,25]. In the present work, we used B_2_-deficient mice to assess the potential contribution of kinin receptors for the clinical course of disease, leukocyte recruitment, and modulation of chemokines expression in the CNS after EAE induction by MOG_35–55_.

## Methods

### Animals

Mice B_2 _knockout (B_2_^-/-^) mice were generated as previously described [24]. Knockout female C57BL/6 X sv129 mice (9–11 wks) and their wild-type (WT) littermate controls were housed under standard conditions and had free access to commercial chow and water. All procedures described in this study had prior approval from the local Ethics Committee that governs animal care and use in research.

### EAE induction

B_2_^-/- ^and WT mice were immunized subcutaneously at the base of the tail with an emulsion containing 100 μg MOG_35–55 _peptide (MEVGWYRSPFSRVVHLYRNGK; (Dept Biophysics, Escola Paulista de Medicina, SP, Brazil) in Freund's complete adjuvant (CFA, Sigma) supplemented with 4 mg/mL *Mycobacterium tuberculosis *H37RA (Difco Laboratories). Pertussis toxin (Sigma), was injected (300 ng/animal, i.p.), on the day of immunization and 48 h later. Animals were monitored daily and neurological impairment was quantified on an arbitrary clinical scale and presented as mean clinical disease severity. Scores were: 0 = no clinical signs, 1 = tail paralysis (or loss of tail tone), 2 = tail paralysis and hind-limb weakness, 3 = hind-limb paralysis, 4 = complete hind-limb paralysis and front limb weakness [26]. The mice were weighed pre- and 14 days post-immunization, the peak of the disease [18]. "Sham animals" refers to WT animals without EAE induction.

### Intravital microscopy in mouse brain

Intravital microscopy of cerebral microvasculature was performed as previously described [18,27]. Briefly, the mice were anaesthetized by intraperitoneal injection of a mixture containing ketamine (150 mg/kg) and xylazine (10 mg/kg). The tail vein was cannulated for administration of fluorescent dyes. A craniotomy was performed using a high-speed drill (Dremel, USA) and the dura matter was removed to expose the underlying pial vasculature. Throughout the experiment, the mouse was maintained at 37°C with a heating pad (Fine Science Tools Inc., Canada) and the exposed brain was continuously superfused with artificial cerebrospinal fluid buffer at pH 7.4, containing in mmol/L: NaCl 132, KCl 2.95, CaCl_2 _1.71, MgCl_2 _0.64, NaHCO_3 _24.6, dextrose 3.71 and urea 6.7, at 37°C.

To assess leukocyte-endothelium interactions, leukocytes were fluorescently labeled by administration of rhodamine 6G (i.v., 0.5 mg/kg body weight) and observed using a microscope (Olympus B201, X20 objective lens, corresponding a 100 μm of area) outfitted with a fluorescent light source (epi-illumination at 510–560 nm, using a 590 nm emission filter). A silicon-intensified camera (Optronics Engineering DEI-470) mounted on the microscope projected the image onto a monitor (Olympus). Rolling leukocytes were defined as the number of white cells moving at a velocity lesser than that of erythrocytes cells. Leukocytes were considered adherent to the venular endothelium if they remained stationary for 30 seconds or longer.

### Histopathology

Brains, cerebellum and the spinal cords were removed just after intravital microscopy. Transverse slices of brain, spinal cord and cerebellum were fixed by immersion in 10% formalin and processed for paraffin embedding. The sections (6.0 μm) were stained with H&E and examined in an Olympus BX51 microscope. Documentation was performed by a coupled digital camera and imaging capture software (Megacybernetics Pro-Express version 4.1 and cool-snap kit).

### Measurement of chemokines

Brain tissue extracts were obtained from control and EAE mice (WT and B_2_^-/-^). Brains were removed after intravital microscopy, and the left and right hemispheres were stored on ice. Thereafter, using Ultra-Turrax, each hemisphere was homogenized in extraction solution (100 mg of tissue per 1 mL) containing 0.4 M NaCl, 0.05% Tween 20, 0.5% BSA, 0.1 mM phenylmetilsulfonil fluoride, 0.1 mM benzetonio chloride, 10 mM EDTA and 20 KIU aprotinin. The brain homogenate was spun at 10000 × g for 10 min at 4°C and the supernatants were stored at -70°C. The concentrations of CCL2, CCL3, CCL5, TNFα and IFNγ were determined in the supernatants of the brain extracts, at a 1:3 dilution in PBS containing 1% BSA, using an ELISA set-up commercially available according to the procedure supplied by the manufacturer (R&D Systems, Minneapolis, MN and Pharmingen, San Diego, CA).

### Statistical analysis

Data are shown as mean ± SEM. Significance was assessed using an ANOVA parametric test with Bonferroni correction for multiple comparisons. Statistical significance was set at P < 0.05.

## Results

### Clinical assessment and histopathology

**Figure 1:**
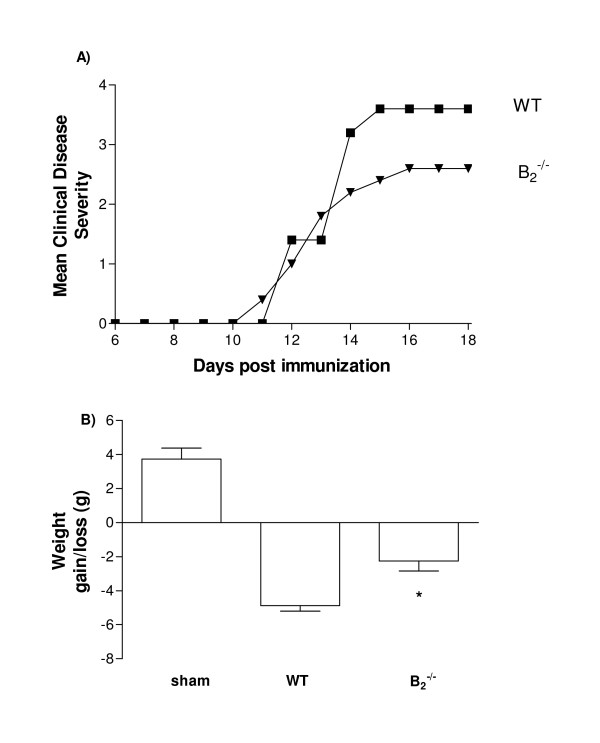
**Clinical assessment of EAE.** Clinical signs (n = 7 mice/group) were daily monitored, comparing B_2_^-/- ^(▼) and WT (■) mice during 18 days after EAE induction (A) and body weight gain or loss (B). Data is expressed as mean ± SEM. *P < 0.05 for B_2_^-/- ^versus WT on the peak of disease (day 14 post-induction).

All WT and B_2_^-/- ^mice developed EAE after MOG_35–55 _administration. Clinical symptoms appeared on day 11 post MOG-injection and peaked around day 14 after which were stable through day 18, the last day for animal evaluation. There were no deaths during the course of EAE development. However, B_2_^-/- ^mice showed a modest but statistically significant reduction (n = 7, mean clinical severity score = 2.4 ± 0.6) in neurological impairment at the peak of disease (day 14) and during the final plateau phase of disease when compared with their WT group counterparts (n = 7, mean clinical severity score = 3.6 ± 0.24) (Figure 1A). In addition, there was significant weight loss in EAE WT animals compared to the EAE B_2_^-/- ^group (Figure 1B).

**Figure 2:**
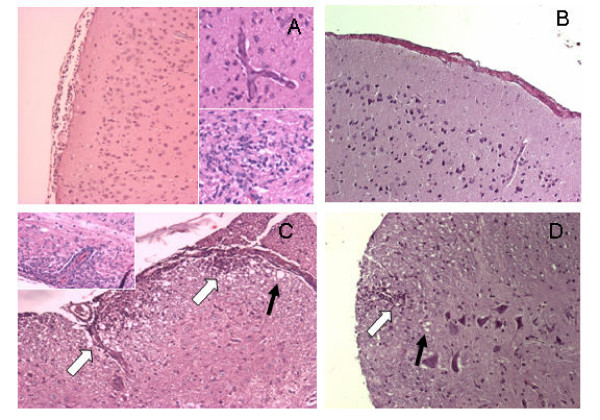
**Histopathological analysis of EAE WT(A, C) and EAE B_2_^-/- ^mice (B, D).** The analysis was performed on H&E-stained sections of brain (A, B) and spinal cord (C, D) at day 14. Observe the marked perivascular infiltration of mononuclear cells in WT brain meninges (A) and the perivascular and inflammatory infiltration of parenchyma (superior and inferior details in A) and spinal cord (C, white arrows). Degenerative changes of white matter are increased in the spinal cord of WT mice in comparison to B_2_^-/- ^mice (C, black arrows). Original magnification 150×.

Histopathological evaluation of the brain, cerebellum and spinal cord was performed in WT and B_2_^-/- ^mice at day 14 after EAE induction. Brains of EAE WT mice showed a marked perivascular infiltration of mononuclear cells around pial and cerebral cortex venules (Figure 2A). The inflammatory process extended from the meninges into cerebral cortex parenchyma and was characterized by focal intense mononuclear infiltration. The meninges and white matter of spinal cord of the EAE WT group presented an intense focal inflammatory response associated with white matter vacuolization (Figure 2C). However, the EAE B_2_^-/- ^group showed only discrete focal inflammatory infiltrates of mononuclear cells, predominantly in association with discrete white matter vacuolization (Figure 2B and 2D).

### Rolling and adhesion of leukocytes in cerebral microvasculature on day 14

**Figure 3:**
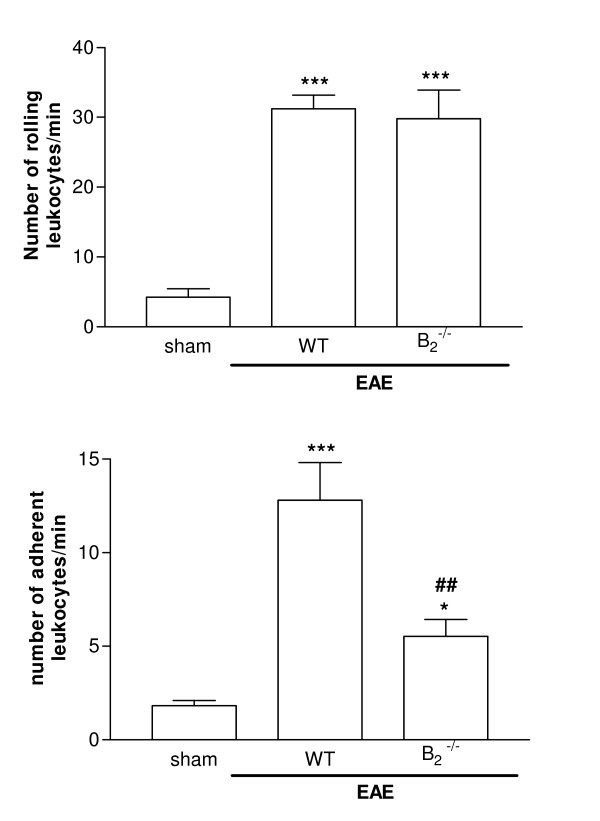
**Leukocyte-endothelial interactions in pial vasculature.** Intravital microscopy was used to assess leukocytes rolling (A) and adhesion (B) on day 14 post-immunization. The analysis was performed in 4–5 vessels per animal (n = 6 per group). Results are expressed as mean ± SEM. ***P < 0.001 when compared to control; ^###^P < 0.001 when compared to EAE WT mice.

EAE pathogenesis requires migration of activated T cells from peripheral lymphoid tissue to the CNS, which presumably occurs in a multi-step manner. To migrate into sites of inflammation, leukocytes must first tether and roll along the vessel before they firmly adhere and emigrate out of the vasculature. Firm adhesion is triggered by the action of chemoattractant molecules, such as chemokines [26]. EAE induced an increase in leukocyte rolling which presented the same profile in all groups. The deletion of B_2 _genes did not alter the rolling of leukocytes on pial vessel walls when compared to WT group (Figure 3A). EAE also induced an increase of leukocyte adhesion in WT mice (Figure 3B). On the other hand, leukocyte adhesion was diminished in B_2_^-/- ^in comparison with WT mice (Figure 3B). In summary, both leukocyte recruitment and histopathological alterations decreased in B_2_^-/- ^when compared to the EAE WT.

### Chemokine release in the brain

**Figure 4:**
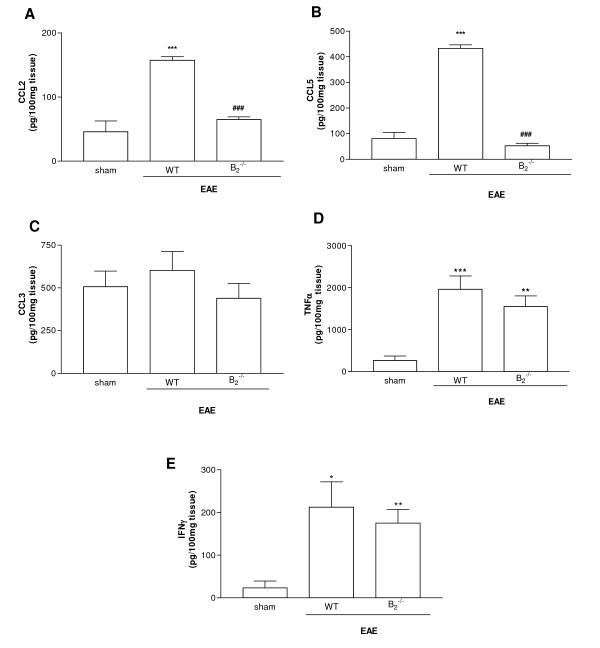
**Kinetics of cytokine and chemokine production in the CNS of EAE mice.** Cerebral levels of CCL2 (A), CCL5 (B), CCL3 (C), TNFα (D) and IFNγ (E) were measured by ELISA (n = 6). Statistically significant differences are indicated by: **P < 0.01 when compared with control; ^##^P < 0.01 when compared with WT.

In order to assess the involvement of kinin receptors in the cerebral chemokine release after EAE induction, brain tissues from WT and B_2_^-/- ^were examined. EAE induction enhanced cerebral production of the chemokines CCL2 and CCL5 in WT mice (Figure 4A, B). In B_2_^-/- ^mice, the levels of CCL2 were significantly lower than those found in WT mice (Figure 4A). In addition, the production of CCL5 was lower in B_2_^-/- ^mice when compared to their WT controls subjected to EAE (Figure 4B). There was no difference in cerebral CCL3 expression in WT mice before or after EAE induction (Figure 4C). Moreover, expression of CCL3 was similar in WT and gene-deficient mice subjected to EAE (Figure 4C).

Considering the important participation of IFNγ and TNFα in T-cell trafficking to CNS, we evaluated the role of kinin receptors on the cerebral production of these pro-inflammatory cytokines. EAE WT and EAE B_2_^-/- ^mice showed increased TNFα and IFNγ in brain tissue, when compared to the control group (Figure 4D, E). However, no statistical difference was found in the cerebral cytokines levels between WT EAE and B_2_^-/- ^EAE mice.

## Discussion

The kallikrein-kinin system is one of the first inflammatory pathways activated after tissue damage. Particularly because of its ability to increase vascular permeability, the kallikrein-kinin system has been investigated for its ability to promote brain edema and secondary brain damage in various models of central nervous system injuries, for example; bacterial meningitis [28], traumatic brain and spinal cord injury [29-32], and global cerebral ischemia [4]. Although, kinins are known to be released and have several effects in the CNS, their cerebral effects in EAE pathogenesis remain unclear.

It is known that pretreatment with B2 receptor antagonist markedly inhibits inflammatory responses in lungs and intestine [15,33] by suppressing leukocyte recruitment and activation in acute models of inflammation [14,34,35]. In addition, treatment with a B2 receptor antagonist decreases CD44, CD54, CD11a and CD11b expression in various cell types after arthritis induction in Lewis rats [36].

This study was designed to provide further insights into the molecular mechanisms of proinflammatory cell trafficking into the CNS in a murine model of EAE. As kinin receptors appear to mediate leukocyte-endothelium interaction induced by certain inflammatory stimuli [4,13], our aim was to determine the putative role of kallikrein-kinin system in the cerebral inflammatory response, via the kinin B2 receptor, after EAE development. For this, we used wild-type (WT) and kinin B2 receptor knockout (B_2_^-/-^) mice subjected to a MOG_35–55_-induced EAE model of multiple sclerosis.

The MOG peptide can induce typical EAE disease in C57BL/6 mice and in other strains. MOG, which represents only ~0.05% of myelin proteins, elicits a major antibody response that has been correlated with disease severity and demyelination in both human and animal models of MS [37,38]. There is some evidence for activation of the kallikrein-kinin system during inflammation in this model [39]. In our hands, mice that lack the kinin B_2 _receptor and are subjected to EAE had a statistically significant reduction in neurological impairment at the peak of disease (day 14) and during the final plateau phase of disease, when compared with the WT EAE group. Our results also reveal lesser degrees of weight loss in EAE B_2_^-/- ^mice when compared to WT EAE animals. These findings are consistent with a previous study that showed prolonged survival, improved motor function, and smaller cerebral infarcts in B_2_^-/- ^after experimental stroke [40]. However, leukocyte recruitment was not investigated in this study. On the other hand, our findings of improvement of neurological clinical score in B_2_^-/- ^mice associated with impaired leukocyte recruitment parameters are in contrast to the findings of Xia et al. [41], which provide evidence for a protective role of the kinin B_2 _receptor against ischemic stroke. In the latter study, the authors also found increased neutrophil accumulation in infarcted brain areas in B_2_^-/- ^mice. Recently these results have been consistently contested, leading to the conclusion that the deficiency of bradykinin receptor B2 is not detrimental in experimental stroke [42], and may be beneficial in other pathological conditions.

EAE pathogenesis requires the infiltration of leukocytes into brain parenchyma. In a previous study undertaken by our group, we showed that there are increased leukocyte-endothelial cell interactions (rolling and adhesion) in brains of EAE mice, as assessed by cerebral intravital microscopy [18]. In the present series of experiments, leukocyte adherence, but not rolling, was suppressed in B_2_^-/- ^mice submitted to the EAE protocol.

The latter findings were corroborated by histopathological examination of brain sections, which showed inhibition of mononuclear cell influx and inflammatory lesions on B_2_^-/- ^mice after EAE induction. In addition, histopathological analysis of spinal cord for EAE WT mice showed a marked inflammatory response with intense vacuolization in white matter, contrasting with very discrete infiltrates and degenerative changes in B_2_^-/- ^mice after EAE induction. Thus, the present study clearly suggests an important role for B_2 _in mediating leukocyte adherence to vessels in brain, and extensively in spinal cord, as suggested by histology of both sites in the same animal.

These suggestions draw support from described patterns of kinin receptor distribution in the CNS. High-density kinin binding sites have been identified in the CNS, mainly in cerebral blood vessels [2]. Previous studies have demonstrated the presence of B_1 _receptor in the brain and spinal cord in different species [43]. In addition, B_2 _receptors have been localized in experimental brain tumors [44]. Chen et al have also shown that the B_2 _receptor is widely expressed within rat brain [45]. In contrast to available knowledge on bradykinin B_2 _receptors in brain, the distribution of these receoptors in spinal cord and the potential functional roles in spinal cord remain unclear. RT-PCR studies reveal the presence of B_1 _and B_2 _receptor mRNA in neurons of dorsal root ganglia in mice [46]. Murone et al. have demonstrated the distribution of B_2 _receptors in the brain and spinal cord of guinea pig and sheep by autoradiography [47,48]. Further studies using intravital microscopy of spinal cord vessels are necessary in order to define functional roles of these receptors in pathological conditions.

Activation of kinin receptors may underlie production of chemokines in several models of inflammation, including response to the cytokine IL-1β in mesenteric venules [49], inflammatory pain [50,51], and allergic rhinitis [21]. Considering previous data, it may be possible that kinin receptors could be regulating the expression of chemokines and, consequently, leukocyte trafficking after EAE induction. Our previous studies showed that the chemokines CCL2 and CCL5, but not CCL3, are involved in the adherence, but not rolling, of leukocytes in the microvasculature of EAE mice [18]. In this study we demonstrate that CCL5 expression is greatly suppressed in EAE B_2_^-/- ^mice, when compared to EAE WT. These findings could partially explain the inhibition of leukocyte adherence observed in B_2_^-/- ^mice, as a probable consequence of decreased levels of CCL5.

The chemokine CCL2 and its receptors CCR2 are thought to play an important role in the pathogenesis of EAE in several conditions. For example, both CCL2 and CCR2 are expressed in brains of patients with MS [52,53] and deletion of CCR2 leads to an almost complete inhibition of MOG_35–55_-induced EAE in mice [16]. Blocking of the CCL2-CCR2 axis with neutralizing antibodies limits the development of subsequent relapses [54]. Our previous studies have also shown a role for CCL2 in leukocyte adherence during murine EAE [18]. In the present experiments, CCL2 levels were lower in B_2_^-/- ^mice than in WT mice after EAE induction. Previous studies have shown that CCL2 may be critical not only for leukocyte recruitment but also for the activation of T cells during inflammatory and immune responses [53,55]. Thus, it is possible that the inhibition of CCL2 production observed in B_2_^-/- ^mice would lead to lesser activation of infiltrated leucocytes and, hence, stabilization of the severity of clinical signs.

There are many studies supporting the clinical involvement of TNF-α in EAE. Elevated expression of TNF-α can be found in the CNS during acute episodes of disease, and blockage of TNF-α with neutralizing antibodies and soluble receptors will ameliorate signs of EAE [56,57]. In order to evaluate the role of the B_2 _receptor in cerebral TNF-α expression after EAE induction, brain tissue extracts were obtained from control, WT EAE and EAE B_2_^-/- ^mice. We found a significant increase in TNF-α levels at day 14 (peak of disease) in both WT and B_2_^-/- ^compared to control mice. Absence of B_2 _receptor did not modulate TNF-α expression after EAE induction. These results are consistent with those of Cunha et al. [58] who demonstrated that B_2 _kinin receptor antagonist, at a dose that inhibits carrageenin-induced hypernociception, does not inhibit release of TNF-α [54]. Direct, bradykinin-independent, release of cytokines was also suggested in rats after administration of large doses of LPS [59].

B_2 _is partially necessary for B_1 _receptor induction as supported by the consistent finding of B_2 _participation in B_1 _mRNA and protein induction in several models of inflammation [60]. The expressed B_1 _is relevant to the control of CCL5 production and to ensuing leukocyte adherence and tissue inflammation. In addition to these B_1_-dependent effects, B_2 _receptor activation is necessary for the production of CCL2 in brains of EAE mice. Previous studies using rodent models of traumatic brain injury have corroborated the finding that B_2 _receptor is present and involved in cerebral alterations [61,62]. Our study clearly demonstrates that blockade of B_2 _receptors is beneficial in EAE mice in reducing inflammatory events such as leukocyte adhesion and activation. We should note, however, that various studies have demonstrated an important role for adhesion molecules in both EAE and MS pathogenesis [63-66] and, at this time, we can not yet exclude the possibility that there is altered expression of adhesion molecules such as ICAM-1 and VCAM-1 on brain endothelial cells of B_2 _deficient animals.

## Conclusion

Our study proposes an important and previously unappreciated involvement of the kallikrein-kinin system in the pathogenesis of EAE. In summary, our results clearly show a definite role for B_2 _receptors in mediating leukocyte adherence, chemokine production and clinical disease outcome. Altogether, our data indicate that blockade of kinin receptors, especially B_2_, may represent an additional and novel therapeutic strategy for the treatment of MS.

## List of abbreviations

ANOVA: analysis of variance; BK: bradykinin; BSA: bovine serum albumine; CFA: complete Freund adjuvant; CNS: central nervous system; CCL: chemokine ligand; CCR: chemokine receptor; ELISA: enzyme linked immunosorbent assay; ICAM: intercellular adhesion molecule; IFNγ: interferon gama; IL-1β: interleukin-1β; MCAO: middle cerebral artery occlusion; MOG: myelin oligodendrocyte glycoprotein; MS: multiple sclerosis; PBS: phosphate buffer sodium; SEM: standard error mean; TNFα: tumor necrosis factor alpha; VCAM: vascular cell adhesion molecule; WT: wild type.

## Competing interests

The authors declare that they have no competing interests.

## Authors' contributions

ACS, MMT and JCT conceived the study, and wrote the manuscript. ACS carried out the experiments and analyzed the results, with the following exceptions: LJ synthetize the peptide MOG. ER extracted RNA samples and prepared cDNA from the brain. RMEA helped in the histological studies. JBP, JLP and MB provided B_2_^-/- ^animals. All authors read and approved the final manuscript.
